# Preclinical Testing of an Oncolytic Parvovirus in Ewing Sarcoma: Protoparvovirus H-1 Induces Apoptosis and Lytic Infection In Vitro but Fails to Improve Survival In Vivo

**DOI:** 10.3390/v10060302

**Published:** 2018-06-03

**Authors:** Jeannine Lacroix, Zoltán Kis, Rafael Josupeit, Franziska Schlund, Alexandra Stroh-Dege, Monika Frank-Stöhr, Barbara Leuchs, Jörg R. Schlehofer, Jean Rommelaere, Christiane Dinsart

**Affiliations:** 1 Division of Tumor Virology, Program Infection, Inflammation and Cancer, German Cancer Research Center (DKFZ), Im Neuenheimer Feld 242, Heidelberg 69120, Germany; z.kis10@imperial.ac.uk (Z.K.); rafael.josupeit@googlemail.com (R.J.); Franziska.Schlund@med.uni-heidelberg.de (F.S.); a.dege@dkfz-heidelberg.de (A.S.-D.); b.leuchs@dkfz-heidelberg.de (B.L.); joteres@arcor.de (J.R.S.); j.rommelaere@dkfz-heidelberg.de (J.R.); c.dinsart@dkfz-heidelberg.de (C.D.); 2 Present address: Department of Pediatrics, Karlsruhe Municipal Hospital, Moltkestraße 90, 76133 Karlsruhe, Germany; 3 Faculty of Engineering, Department of Chemical Engineering, Imperial College London, South Kensington Campus, London SW7 2AZ, UK; 4 Division of Viral Transformation Mechanisms, Program Infection, Inflammation and Cancer, German Cancer Research Center (DKFZ), Im Neuenheimer Feld 242, 69120 Heidelberg, Germany; frank-stoehr.monika@t-online.de

**Keywords:** Ewing sarcoma, protoparvovirus H-1 (H-1PV), oncolytic virus, lytic infection, apoptosis

## Abstract

About 70% of all Ewing sarcoma (EWS) patients are diagnosed under the age of 20 years. Over the last decades little progress has been made towards finding effective treatment approaches for primarily metastasized or refractory Ewing sarcoma in young patients. Here, in the context of the search for novel therapeutic options, the potential of oncolytic protoparvovirus H-1 (H-1PV) to treat Ewing sarcoma was evaluated, its safety having been proven previously tested in adult cancer patients and its oncolytic efficacy demonstrated on osteosarcoma cell cultures. The effects of viral infection were tested in vitro on four human Ewing sarcoma cell lines. Notably evaluated were effects of the virus on the cell cycle and its replication efficiency. Within 24 h after infection, the synthesis of viral proteins was induced. Efficient H-1PV replication was confirmed in all four Ewing sarcoma cell lines. The cytotoxicity of the virus was determined on the basis of cytopathic effects, cell viability, and cell lysis. These in vitro experiments revealed efficient killing of Ewing sarcoma cells by H-1PV at a multiplicity of infection between 0.1 and 5 plaque forming units (PFU)/cell. In two of the four tested cell lines, significant induction of apoptosis by H-1PV was observed. H-1PV thus meets all the in vitro criteria for a virus to be oncolytic towards Ewing sarcoma. In the first xenograft experiments, however, although an antiproliferative effect of intratumoral H-1PV injection was observed, no significant improvement of animal survival was noted. Future projects aiming to validate parvovirotherapy for the treatment of pediatric Ewing sarcoma should focus on combinatorial treatments and will require the use of patient-derived xenografts and immunocompetent syngeneic animal models.

## 1. Introduction

In pediatric cancer patients, tumors of the Ewing sarcoma (EWS) family are the second most frequent group of diseases of the skeletal system. Yearly, about 2.9 new cases of Ewing sarcoma are diagnosed per million persons below the age of 19 [1]. Two groups of Ewing sarcoma patients have an especially poor outcome. Firstly, patients with primary metastatic Ewing sarcoma have a 5-year survival rate below 30%. Secondly, the long-term survival of relapse patients is reported to be less than 10%. Thus, for patients with metastasizing or recurring Ewing sarcoma, more effective treatment options are needed to improve the dismal prognosis of their disease [2].

Alongside the first approval of an oncolytic virus for the treatment of adult melanoma patients by the US food and drug administration, both preclinical and clinical research on oncolytic viruses have been intensified in pediatric oncology [3]. Few oncolytic viruses, however, have been reported to be effective against Ewing sarcoma in in vitro or in vivo preclinical evaluations. So far, only three oncolytic viruses have been described to show antineoplastic efficacy against pre-established human Ewing sarcoma in xenograft models: oncolytic HSV [4], Seneca Valley virus [5], and Reovirus [6]. On the basis of the data produced, several clinical trials have been initiated and are still ongoing [7].

In the present study we have performed systematic preclinical in vitro and in vivo assessments of protoparvovirus H-1 (H-1PV) for the treatment of pediatric Ewing sarcoma. H-1PV is a wild-type rodent parvovirus shown in vitro and in preclinical tumor models to have antineoplastic effects against a broad range of human cancers, including glioblastoma, pancreatic adenocarcinoma, breast cancer, and Burkitt lymphoma [8]. Parvoviral particles are non-enveloped and contain a single-stranded DNA genome of about 5100 nucleotides, with one open reading frame (ORF) encoding the two non-structural proteins (NS1, NS2) and another encoding the two capsid proteins (VP1, VP2) [9] and the non-structural, small alternatively translated (SAT) protein [10]. Parvovirus H-1PV triggers several distinct cell death processes, including apoptosis and the cytosolic relocation and activation of lysosomal cathepsins [11]. As parvoviruses are among the smallest viruses, they easily circulate between different compartments. As in a comparative in vitro study the standard wild-type H-1PV virus displayed higher cytotoxicity towards osteosarcoma cells than two mutant variants [12], we have chosen to focus first on this standard wild type. We have analyzed the transduction efficiency, replication, and lytic activity of H-1PV in four Ewing sarcoma cell lines with different p53 mutation statuses (Table 1). We provide evidence that this oncolytic protoparvovirus exerts cytotoxic effects on these cells in vitro. Although virus-triggered repression of tumor proliferation was also observed in a Ewing sarcoma xenograft model, this in vivo antineoplastic effect failed to reach statistical significance and to translate into improved survival.

## 2. Materials and Methods

### 2.1. Sources and Culture Conditions for Human Cell Lines

Primary human osteoblasts were provided by PromoCell GmbH (Heidelberg, Germany). The four Ewing sarcoma cell lines used are commercially available and described in Table 1. They were purchased from the following companies or institutions: SK-ES-1 from CLS (Cell Lines Service GmbH, Eppelheim, Germany); CADO-ES, MHH-ES, and TC-71 from DSMZ (Leibnitz-Institut DSMZ—Deutsche Sammlung von Mikroorganismen und Zellinien GmbH, Hannover, Germany).

All cell lines were cultured under standard conditions at 37 °C and under 5% CO_2_ on media supplemented with 100 U/mL penicillin and 100 µg/mL streptomycin (final concentrations) in an incubator with a humidified atmosphere. Non-transformed human osteoblasts were grown in Osteoblast Growth Medium (PromoCell GmbH, Heidelberg, Germany). The CADO-ES and MHH-ES cell lines were grown in RPMI 1640 medium supplemented with 2 mM l-glutamine and 10% fetal bovine serum. SK-ES-1 was grown in McCoy’s 5a medium supplemented with 1.5 mM l-glutamine and 15% fetal bovine serum (FBS). TC-71 cells were passaged in Iscove’s MDM medium supplemented with 10% FBS. For passaging, cells were typsinized with either 0.05% or 0.25% Trypsin-EDTA solution and then resuspended in fresh culture medium. All cell lines were routinely tested for microbial contamination and genomic identity with the Multiplex human Cell line Authentication test (MCA) available from a specialized service provider (Multiplexion GmbH, Friedrichshafen, Germany) [13].

### 2.2. Production of Infectious Viral Particles

Wild-type H-1 protoparvovirus (H-1PV) was produced on NBK-324K human embryonic kidney cells as published previously [14] at the Virus Production & Development Unit, Division of Tumor Virology, German Cancer Research Center, Germany. Briefly, NBK-324K cells were infected at a multiplicity of infection (MOI) of 10^−2^ plaque-forming units (PFU) per cell and allowed to replicate the virus for four to five days. The cells and supernatant were then harvested, filtered (maximal particle diameter: 0.2 µm), and subsequently purified by iodixanol gradient centrifugation. This procedure decreased contaminating endotoxins to below 2.5 EU/mL in the virus stocks.

### 2.3. Detection of Infectious H-1PV Particles

After virus production, viral titers were determined in triplicate by plaque assay according to standard protocols [15]. For measuring viral titers in the supernatants of the Ewing sarcoma cell cultures, infected cell dot blot hybridization assays were applied as previously described [14]. Briefly, NB-324K human newborn kidney cells (7.6 × 10^3^ cells/well) were seeded into 96-well plates. After 24 h, they were infected with 10-fold serial dilutions of the virus sample and incubated for 72 h at 37 °C under 5% CO_2_ and at saturating humidity. Next, alkaline lysis of the virus suspension was performed with 0.75 M NaOH and the DNA was transferred to a nylon membrane and cross-linked. After hybridization with an NS1-specific ^32^P-labeled probe and subsequent washing steps, X-ray films were exposed to the radioactive blots for autoradiography. The MOI used in each experiment is indicated in plaque forming units (PFU) or infectious units (IU) in the corresponding figure.

### 2.4. Extraction of H-1PV DNA and Qantification by Real-Time PCR

Cell culture media were collected from cell culture dishes of adherently growing cell lines at regular time intervals after infection. To release viral DNA from virions, one-tenth of the total volume of this supernatant (i.e., 1 mL) was subjected to alkaline lysis in 1 M NaOH in TE buffer for 30 min at 56 °C. After stopping the reaction by neutralization with HCl, the samples were diluted to 1:100 in sterile water. Quantification of viral DNA in these solutions was carried out by real-time qPCR with an NS1-specific TaqManTM probe (5′-6-FAM-ATGCAGCCAGACAGTTA-Q-MGB 3′) from Applied Biosystems (Foster City, CA, USA) according to previously published procedures. The primers used were NS1-FOR: 5′-GCGCGGCAGAATTCAAACT-3′, NS1-REV: 5′-CCACCTGGTTGA GCCATCAT-3′ [16]. Serial dilutions of a plasmid containing the NS1 sequence in the range of 10^1^–10^8^ copies/reaction were applied to allow quantification of the templates under standardized conditions.

### 2.5. Conditions of in vitro Infection Experiments

Adherently growing osteoblasts or Ewing sarcoma cells were infected with H-1PV 12 to 24 h after cell counting and seeding. The infection was carried out in a serum-free medium for 2 h at 37 °C in order to increase the efficiency of adhesion to the cells. The medium was then supplemented with the FBS required for optimal growth of the cell line concerned.

### 2.6. Microscopy of H-1P-Infected Cells

A Keyence BZ 9000 microscope (KEYENCE Microscope Europe NV/SA, Mechelen, Belgium) and the imaging software BZ II Viewer and BZ II Analyzer supplied by the manufacturer were used to document the morphology of infected cells after crystal violet staining.

### 2.7. Analysis of the Cell Cycle, Including Sub-G1 Apoptotic Cells, during H-1PV Infection

A total of 10^6^ Ewing sarcoma cells were seeded into each 10-cm-diameter dish and infected with H-1PV at 1 PFU/cell and cultured until the cells reached confluence (after 72 to 120 h). Mock-infected cells were harvested at the corresponding time points to serve as controls. Cells were washed twice with PBS and then fixed with 0.7 mL ice-cold 100% ethanol and 0.2 mL PBS. After fixation, the cells were stored at 4 °C for up to 10 days before further processing. Before FACS analysis the cells were pelleted for 10 min at 400× *g* and 4 °C and washed twice with PBS. Pellets were resuspended in PBS containing 100 mg/mL RNase H and 5 µg/mL propidium iodide (Sigma-Aldrich Inc., St. Louis, MO, USA). The stained cells were filtered through a 41-µm nylon mesh, incubated on ice for 1 hour in the dark, and then analyzed for their DNA content on a FACSort flow cytometer (Becton, Dickinson and Company, Franklin Lakes, NJ, USA). Experiments were performed in triplicate and at least 20,000 events were recorded and analyzed with the Cell-Quest^TM^ software (from Becton-Dickinson).

### 2.8. Quantatitation of Cell Viability and Cell Lysis

Between 1000 and 2000 cells per well were cultured in 96-well plates and infected at the MOIs indicated in the relevant figures. The mitochondrial metabolic activity of the Ewing sarcoma cells was assayed by adding 3-(4,5-dimethylthiazol-2-yl)-2,5-diphenyltetrazolium bromide (MTT) from Sigma-Aldrich^®^, Inc., (St. Louis, MO, USA) to the cells as previously published [17]. Three and six days after infection, 50 µL of the medium were removed and transferred into a second separate 96-well plate to perform the LDH-release assay as described below. After this the cells were incubated with medium containing 0.5 µg MTT per mL. This incubation was stopped when the cytoplasm of the positive control cells was completely stained but no extracellular crystallization of the dye had occurred (maximum incubation time: 2 h). The supernatant was then discarded and the cells were allowed to dry. For the photometric analyses, 100 µL propanol-2 was added to each well and shaken for 30 min, by which time the dye was completely dissolved. It was quantified by measuring the extinction at 570 nm (Multiscan Plus™, Titertek Instruments Inc., Huntsville, AL, USA).

Cell lysis was assayed by measuring the amount of lactate dehydrogenase (LDH) released into the culture medium with the Cytotox 96™ cytotoxicity assay kit according to the manufacturer’s instructions (Promega, Mannheim, Germany). The absorbance at 490 nm of the red formazan generated by the LDH-catalyzed reaction was measured in the above-mentioned microplate reader. Both the cell viability tests and the cell lysis assays were carried out in quintuplicate.

### 2.9. Real-Time Proliferation Measurements

Three thousand Ewing sarcoma cells per well were seeded in a special 96-well plate (E-plate 96™, Roche Applied Science, Mannheim, Germany) and the proliferation index was recorded. Cell proliferation was evaluated at 30-min intervals on the basis of real-time impedance measurements performed with the xCELLigence system (xCELLigence MP™, Roche Applied Science, Mannheim, Germany). Experiments were performed in ten replicates and continued until the mock-treated control cells reached confluence. Dose-response-graphs and the resulting LD50s were calculated by analyzing 10 wells per dose according to the manufacturer’s recommendations.

### 2.10. Animal Experiments

Experiments on animals were conducted according to institutional and legal regulations for animal experimentation, as approved by the Animal Welfare Committee of the German Cancer Research Center and by the land Baden-Württemberg. Four-week-old female Fox NMRI nude mice were subcutaneously injected with 10^6^ TC-71 cells resuspended in 100 µL BD Matrigel™ Basement Membrane Matrix (Beckton Dickinson, Heidelberg, Germany). On day 7 after implantation, all animals showed successful engraftment and were randomly assigned to two groups. Animals in the control group (*n* = 15) received an intratumoral injection of 100 µL 0.9% NaCl solution, whereas the H-1PV-treated animals (*n* = 15) were injected with 100 µL solution containing 10^9^ PFU H-1PV produced under GMP-like conditions.

Tumor size was determined in three dimensions with an electronic caliper every two to three days. The tumor volume was calculated as V = 4/3πabc, where a, b, and c represent the lengths of the semi-axes of the spheroid formed by the xenograft tumor.

### 2.11. Statistical Methods

Mean tumor volumes were calculated for the treatment and control groups and the respective standard errors of the mean are given as error bars. The statistical significance of differences between group tumor-volume means was tested with Student’s *t*-test. The criterion for statistical significance was *p* < 0.05. In order to compare event-free survival distributions between treatment and control groups, the exact log-rank test, as implemented in Sigma Plot^®^ 12.3 (Systat Software Inc., San Jose, CA, USA), was used. *p*-values were two-sided and were not adjusted for multiple comparisons.

## 3. Results

### 3.1. Ewing Sarcoma (EWS) Cells Are Susceptible to H-1PV Infection and Sustain the Synthesis of Viral Proteins

To demonstrate that the major H-1PV viral proteins were selectively produced in infected Ewing sarcoma cells, western blot analyses of the viral proteins NS1, NS2, VP1, and VP2 were performed. NS1 and NS2 are nonstructural proteins required for all steps of the viral life cycle [15]. VP1 and VP2 are structural viral capsid proteins [16]. In osteoblasts used as non-transformed mesenchymal cell controls, the viral NS1, NS2, and VP proteins were not detected. This indicates that these cells are resistant to H-1PV infection.

**Figure 1:**
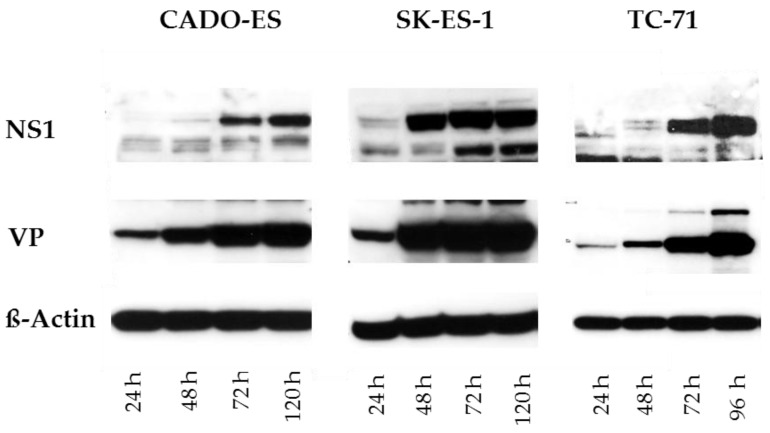
Protoparvovirus H-1 (H-1PV) can infect human Ewing sarcoma cells. At the time points indicated after mock infection or infection with H-1PV at MOI = 1 PFU/cell, cells were collected for protein extraction and western blot analysis. Viral proteins were detected with polyclonal antisera against the viral nonstructural protein 1 (NS1, 100 kD) and the capsid proteins 1 and 2 (VP1, 97 kD, and VP2, 68 kD). Detection of beta-actin (45 kD) was used as the loading control.

In all four EWS cell lines infected with wild-type Protoparvovirus H-1PV at 1 PFU/cell, the viral nonstructural and capsid proteins were detected (shown in Figure 1 for CADO-ES, SK-ES-1, and TC-71). Viral protein levels varied between the EWS cell lines but increased consistently over time and persisted until the end of the observation period, 120 h after infection.

### 3.2. H-1PV Infection Interferes with the Cell Cycle in Ewing Sarcoma Cell Lines

**Figure 2:**
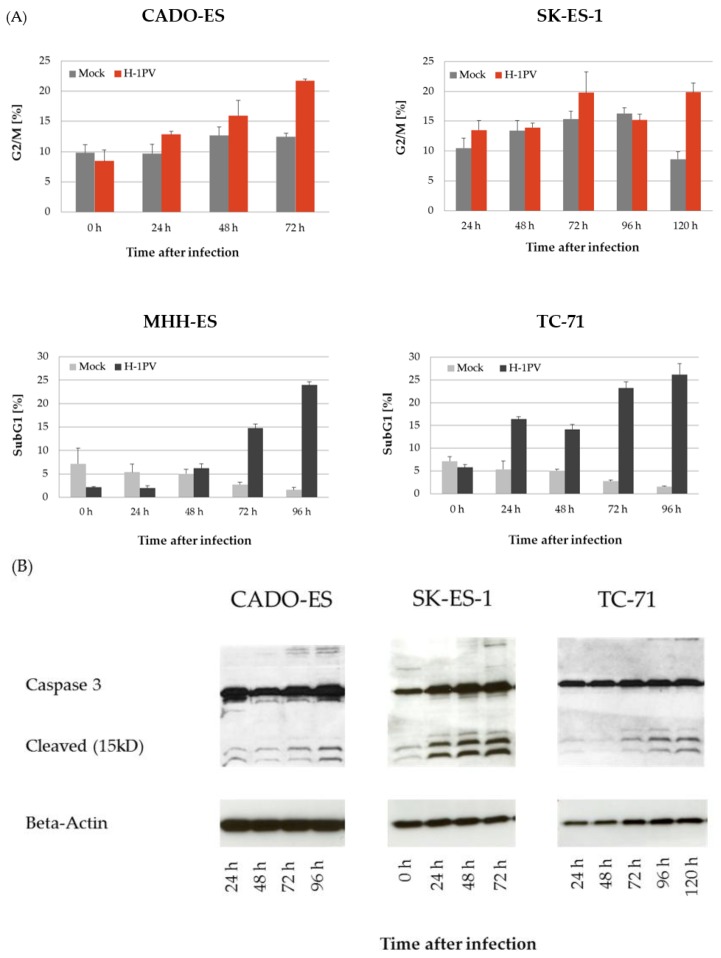
H-1PV infection induces disturbances of the cell cycle in all four Ewing sarcoma cell lines. (**A**) Cells were harvested at the time points indicated after infection with H-1PV at 1 PFU/cell. Propidium iodide staining and flow-cytometric cell cycle analysis were then performed. H-1PV infection induced G2-arrest in the CADO-ES and SK-ES-1 cell lines and the appearance of a sub-G1 population (indicating apoptosis induction) in the MHH-ES and TC-71 cell lines; (**B**) Induction of apoptosis during H-1PV infection was confirmed by western blotting for caspase-3.

The effects of H-1PV infection on the cell cycle were analyzed in the Ewing sarcoma cell lines by propidium iodine staining followed by flow cytometry. As previously described for osteosarcoma cells [12], H-1PV infection induced G2/M-arrest in the Ewing sarcoma cell lines CADO-ES and SK-ES-1 (Figure 2A, upper panels).

In MHH-ES and TC-71 cells, H-1PV infection also led to an accumulation of cells in G2/M within the first 48 h after infection. Subsequently, the proportion of cells arrested in G2/M significantly decreased and a cell population characterized by the presence of sub-G1 DNA content (less than 2n) appeared. Over time a constant increase of this cell population undergoing DNA fragmentation and apoptosis was observed. Ninety-six hours after infection with H-1PV, the fraction of apoptotic cells was about 25% in both Ewing sarcoma cell lines (Figure 2A, lower panels). In keeping with the results of the cell cycle analysis, no significant caspase-3 activation was observed in CADO-ES cells. Along with increasing apoptotic DNA fragmentation, a constant increase in activated (i.e., cleaved) caspase-3 was detected by western blot analysis in MHH-ES, SK-ES-1, and TC-71 cells (shown for SK-ES-1 and TC-71 in Figure 2B).

### 3.3. H-1PV-Induced Cytotoxic Effects in Ewing Sarcoma Cell Lines Correlate with the Dose of Input Virus

**Figure 3:**
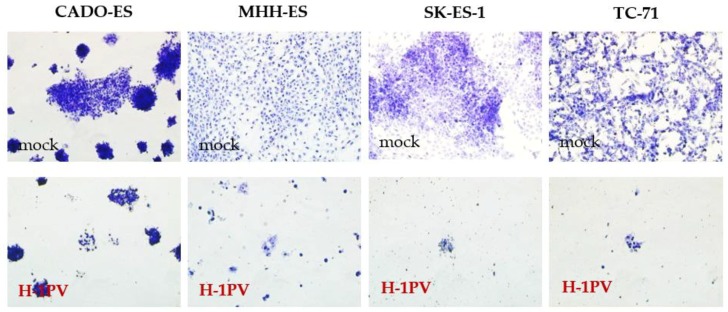
Cytopathic effects of H-1PV infection in Ewing sarcoma cells. Pictures of cells seeded in 96-well plates were taken after staining with crystal violet five days after infection with wild-type Protoparvovirus H-1PV at MOI 1. Magnification: 10×.

To determine the dose of input H-1PV virus capable of inducing significant cytopathic effects, all the cell lines were infected with increasing doses of wild-type H-1PV (ranging from 0.01 to 50 PFU per cell). Three days after H-1PV infection, cytopathic effects were visible in all four Ewing sarcoma cell lines. These effects became more obvious over time, as documented on day five after infection (Figure 3).

**Figure 4:**
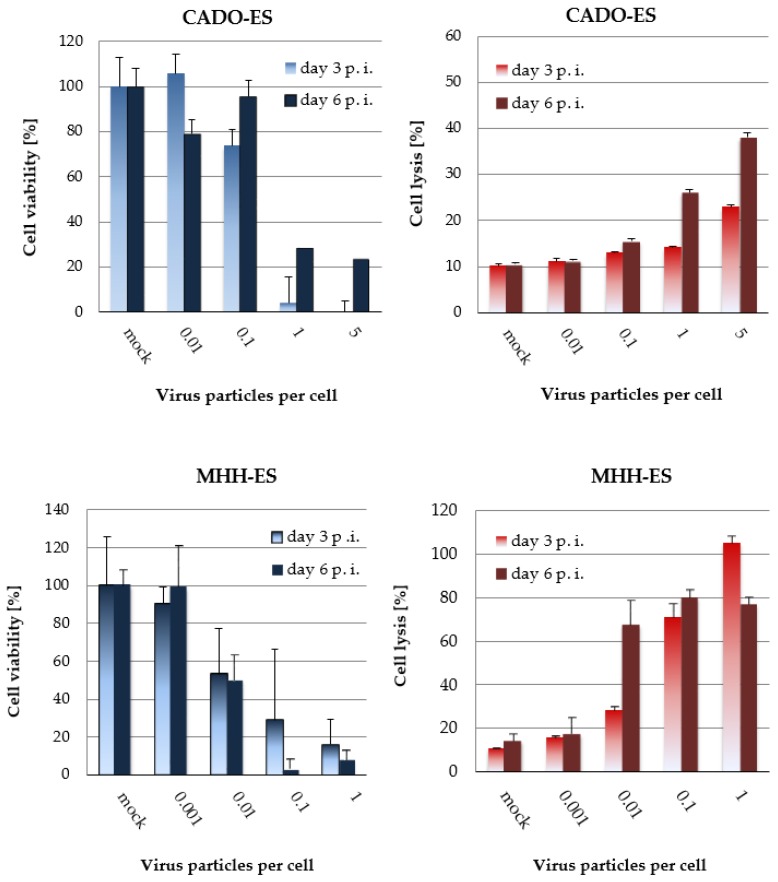
Ewing sarcoma cells undergo lytic infection by H-1PV. The viability of Ewing sarcoma cells was quantified in MTT tests three and six days after infection with wild-type H-1PV at the indicated MOI. Metabolic activity is expressed in relation to mock-treated control cells. Cell lysis due to H-1PV infection was quantified in LDH-release assays. Complete lysis of cells with a detergent-containing lysis buffer was used as a reference. Error bars indicate standard errors of the mean (SEM). These results are also summarized in Table 1.

Virus-induced effects on cell viability were quantified by measuring the activity of mitochondrial metabolism in MTT tests. Dose-dependent cytostatic effects were demonstrated in all four cell lines and are shown for CADO-ES and MHH-ES (Figure 4A, left column). Simultaneously, the capacity of the virus to lyse Ewing sarcoma cells was quantified in LDH-release assays (Figure 4B, right columns). On the basis of these data the LD50 of H-1PV was determined for each cell line and found to vary from 0.01 PFU to 5 PFU per cell (Table 1).

**Figure 5:**
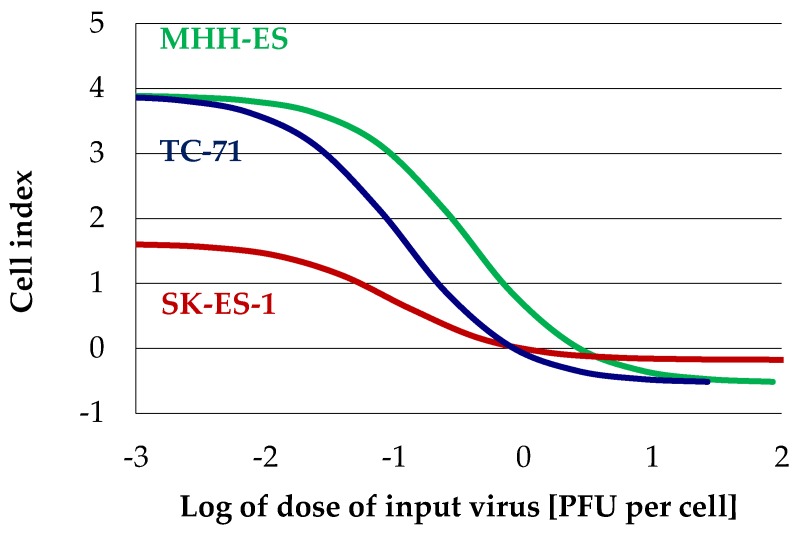
Ewing sarcoma cells undergo lytic H-1PV infection. Dose-response graphs for H-1PV-infected cells, determined by real-time proliferation measurements as described under Materials and Methods. EC50 values were calculated on the basis of these experiments are shown in Table 2.

Three of the four EWS cell lines tested grow as fibroblast-like adherent cultures and could thus be used for real-time proliferation measurements with the xCelligence instrument. The CADO-ES cell line could not be analyzed by this method because of its semi-adherent growth. For MHH-ES, SK-ES-1, and TC-71 cells, virus dose-response graphs were established with high statistical reliability (*R* > 0.9), indicating an ED50 of input virus between 0.1 and 1 PFU per cell (Figure 5 and Table 2).

### 3.4. Ewings’s Sarcoma Cells Efficently Replicate H-1PV

**Figure 6:**
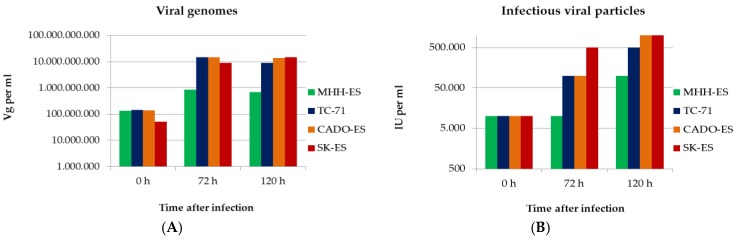
Ewing sarcoma cells are capable of active H-1PV replication. (**A**) Viral genomes in the supernatants of H-1PV-infected Ewing sarcoma cells were quantified by NS1-specific qPCR at the time points indicated; (**B**) Titers of infectious viral particles released into the supernatants of H-1PV-infected cells were quantified by propagation on NBK cells after serial dilution and detection of viral DNA on dot blots with a ^32^P-labeled NS1-specific oligonucleotide probe.

Full permissiveness to a viral infection includes the capacity of the virus to replicate in the host cell. To test the ability of H-1PV to produce infectious viral progeny in Ewing sarcoma cells, a time-course experiment was performed. The viral DNA content and the titer of fully infectious viral particles in the cell culture medium were determined at the indicated time points after infection for each of the four Ewing sarcoma cell lines. After infection with H-1PV at MOI = 1 PFU/cell, viral genome copy titers were determined by real-time PCR in the supernatants of infected cells. A titer increase was taken as indirect evidence of successful replication of viral DNA. The observation period was 120 h. After 72 h, the viral genome titers of the supernatants reached a plateau and did not increase further. MHH-ES cells showed the lowest viral DNA replication capacity: only a 10-fold increase in viral genomes was observed. In CADO-ES, SK-ES-1, and TC-71 cells, in contrast, the number of genome copies in the supernatant increased by two log steps (Figure 6, left panel). In the same time-course experiment, titers of infectious particles were determined by titration on NBK-324K cells. The increase in the titer of fully infectious viral particles in the supernatant was taken as a measure of the efficiency of production of fully infectious viral progeny by the Ewing sarcoma cells. The observed increase ranged from 10-fold in MHH-ES cells to 50-fold in TC-71 cells and 100-fold in CADO-ES and SK-ES-1 cells. In conclusion, efficient virus replication, correlating with infectious particle production, was demonstrated in all four Ewing sarcoma cell lines.

### 3.5. Intratumoral Infection with H-1PV Represses Human Ewing Sarcoma Cell Proliferation in a Xenograft Mouse Model but Fails to Improve Survival

**Figure 7:**
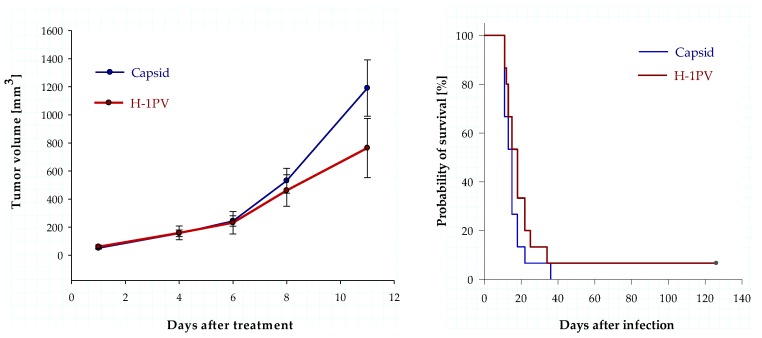
H-1PV infection represses the growth of subcutaneous TC-71 xenograft tumors in mice. Mean tumor volume in animals after a single intratumoral injection with empty, UV inactivated H-1PV capsids (control group, *n* = 15, blue graph). Animals in the treatment group (*n* = 15, red graph) received a single intratumoral injection of 10^9^ PFU wild-type H-1PV. For each group of animals, the standard errors of the mean (SEM) are indicated as error bars. Survival data for the two groups of animals show no statistically significant difference, but indicate one animal in complete long-term remission after H-1PV virotherapy.

Having identified human Ewing sarcoma cells as fully permissive to H-1PV infection in vitro, we next assessed the effects of H-1PV infection on tumor proliferation in vivo. To determine the antineoplastic efficacy of Protoparvovirus H-1PV, a single dose of wild-type H-1PV (10^9^ PFU) was injected into established TC-71 xenograft tumors on day eight after implantation. Volumetric analysis of the tumors showed a difference in mean tumor volume between the control group (treated with an equal dose of purified and UV-irradiated empty capsids) and the H-1PV-treated group (Figure 7, left panel). Eleven days after treatment, the animals treated with empty capsids had a mean tumor volume of 1190 mm^3^, whereas the mean tumor volume in the H-1PV-infected animals was 752 mm^3^; however, this virus-induced repression of tumor cell proliferation did not reach statistical significance (*p* = 0.08).

This single intratumoral H-1PV injection achieved complete remission in one out of fourteen animals. The successfully treated animal remained tumor-free and in good condition for more than four months. In contrast, none of the animals in the control group survived more than 36 days (Figure 7, right panel). Nevertheless, a comparative analysis of survival showed a median survival of 18 days in the control group vs. 15 days in the control group (*p* = 0.197; Logrank-Test), which failed to demonstrate statistical significance.

In an attempt to increase the therapeutic efficacy of intratumoral H-1PV administration, a second TC-71 xenograft experiment was performed. The animals were subdivided into three groups: animals mock-infected with sodium chloride solution, animals receiving a single intratumoral H-1PV injection, and animals receiving repeated injections of the virus twice weekly for as long as they survived. Although repeated injections optimized virus delivery throughout the tumor mass, they did not improve the efficacy of the H-1PV treatment (Supplementary Materials Figure S1). Differences in tumor volume and survival between the control and treatment groups were statistically insignificant.

## 4. Discussion

### 4.1. H-1PV Selectively Infects Transformed Human Cells of Mesenchymal Origin

So far, preclinical and clinical development of protoparvovirus H-1 has mainly focused on epithelial and neuronal tumors. Recently a phase I/IIa clinical trial on recurrent or refractory glioblastoma patients was successfully completed. After intravenous application, a favorable systemic distribution of the virus throughout the body allowed H-1PV to selectively infect the intracranial tumors. After intravenous H-1PV application, no grade III or IV toxicity was observed. Signs of an immune response, such as infiltration of the infected tumors with cytotoxic T-cells, provided first evidence of the capacity of the virus to recruit the immune system of the host to induce an antitumor immune response [18]. On the basis of these promising safety data, a second trial, on pancreatic adenocarcinoma patients, has been initiated [19].

The significant antineoplastic effects of protoparvovirus H-1PV on malignant tumors of mesenchymal origin were already recognized more than three decades ago. In a preventive model, productive H-1PV infection of neonate Syrian hamsters on day six after birth and subsequent seroconversion protected 50% of the infected animals from developing fibrosarcoma induced by the carcinogen 7,12-dimethylbenz(a)anthracene, [20]. To date, however, the therapeutic efficacy of protoparvovirus H-1 on human sarcomas of different types, especially pediatric sarcomas, has not been systematically evaluated in preclinical models. Given the above-mentioned promising historical experiments and the now well-characterized safety profile of this virus, we have performed a systematic preclinical assessment of H-1PV as a therapeutic agent against osteosarcoma [12] and Ewing sarcoma, the most frequent types of bone tumors in children and adolescents.

We have previously shown that mesenchymal cells can be infected by recombinant H-1PV-based viral vectors, but we have also documented that wild-type H-1PV at a dose below 50 PFU/cell has no significant impact on the morphology or viability of neonate fibroblasts or human osteoblasts in short-term culture [12]. In the experiments presented here, we confirm the oncoselectivity of H-1PV infection in mesenchymal cells, since we detected the H-1PV nonstructural and capsid proteins in all the tested Ewing sarcoma cell lines after infection with H-1PV at MOI 1, but not at any point in time in osteoblasts infected at this MOI. On the basis of the in vitro experiments on osteosarcoma cells, we have previously estimated the therapeutic index of wild-type H-1PV infection to range between 5 and 100 [12]. Here, given our previously published toxicity data for non-transformed mesenchymal cells and the cytotoxic doses determined here on Ewing sarcoma cells, the estimated therapeutic index of H-1PV parvovirotherapy for EWS appears even more favorable: between 10 and 5000.

### 4.2. Ewing Sarcoma Cells Are Fully Permissive to Wild-Type H-1PV Infection

In contrast to the vectors used for cancer gene therapy, the self-replicating oncolytic viruses envisaged for cancer therapy can promote successive cycles of lytic infection, allowing the penetrance of a lytic infection throughout an entire tumor mass. Secondary rounds of infection within a tumor require efficient virus replication and viral egress from the infected cells. The first clinical applications of H-1PV in pediatric clinical trials will most likely address malignant diseases for which there is both in vitro and in vivo evidence that the virus can replicate efficiently in the malignant cells and lyse enough of them to induce an antitumor immune response. Although wild-type protoparvovirus H-1PV has been characterized as a self-replicating oncolytic virus, its replication efficiency varies according to the type of tumor cell [21]. The present study is the first systematic preclinical characterization of H-1PV-induced effects on the cell cycle and viability of EWS cell lines and of the capacity of these lines to replicate the virus. We demonstrate that H-1PV can infect all four tested EWS cell lines and that an infection at MOI = 1 PFU/cell is sufficient to induce sustained levels of the main viral proteins for at least one week.

We show that infected EWS cells in culture can replicate H-1PV efficiently, and that this leads to a 100- to 1000-fold infectious particle titer increase in the culture supernatant. We further demonstrate that the viral descendants are fully competent to infect NB-324K cells and that the viral genome copy number increase correlates with infectious particle production in the cell line considered. Thus, in contrast to what was shown for human osteosarcoma cell cultures [12], the propagation of wild-type H 1PV in EWS cells is not hampered by a limited capacity of the host cells to replicate the virus.

The results of our in vitro time-course experiments show that H-1PV can induce virus-specific disturbances of the cell cycle in all four EWS cell lines studied here, although interestingly, the effects observed depended on the cell line tested. CADO-ES and SK-ES-1 cells, which showed the highest input virus LD50 values, remained in G2/M arrest, whereas MHH-ES and TC-71, more susceptible to H-1PV-induced cytotoxic effects, displayed a progressive increase of cells in subG1; i.e., undergoing DNA fragmentation. H-1PV-induced DNA fragmentation was associated with apoptotic cell death, and the induction of apoptosis was confirmed in western blot experiments revealing active, cleaved caspase-3 in the H-1PV-infected cells.

As shown by crystal violet staining, H-1PV causes significant changes in cell morphology and both antiproliferative and direct cytotoxic effects in all four tested EWS cell lines infected at MOI = 1 PFU/cell. Our experiments quantifying cell viability and virus-induced cell lysis in the presence of increasing doses of H-1PV on days three and six after infection clearly illustrate the dose-dependent cytotoxicity of the virus in all four EWS cell lines. The lytic effect of H-1PV in Ewing sarcoma cells was observed at MOIs between 0.01 and 5 PFU per cell and occurred whatever the origin of the cell line. These data were confirmed by real-time proliferation measurement: doses of input H-1PV equal to or below 1 PFU/cell were identified as LD50 doses in the adherently growing EWS cell lines; i.e., one established from a primary tumor, one from a recurrent tumor, and one from a peritoneal metastatic Ewing sarcoma.

### 4.3. Animal Data Show Antiproliferative Effects of H-1PV but Fail to Demonstrate Significant Antineoplastic Efficacy in a Ewing Sarcoma Xenograft Model

We have found a single H-1PV injection into established tumors on day eight after implantation to cause repression of tumor proliferation in a subcutaneous human Ewing sarcoma xenograft model. For this model we selected TC-71, a fully permissive pediatric Ewing sarcoma cell line. In this cell line, viral protein expression, efficient virus replication, and virus-induced cytotoxic effects had been clearly documented in vitro. On day 11, after the initiation of treatment, the mean tumor volume was reduced by more than a third in the group of animals treated with H-1PV, as compared to the control group. Yet no statistically significant improvement of survival was observed in the virotherapy group, although one animal in this group displayed persisting long-term remission. In a second experiment, we tried to increase the number of animals displaying persisting tumor remission by injecting H-1PV twice weekly as a continuous treatment for as long as the animal survived. Contrarily to our expectations, no improvement of in vivo therapeutic efficacy was observed, even though virus administration had been optimized and the cumulative dose of virus delivered had been increased to 5 × 10^9^ PFU per animal, the maximum dose delivered to humans in the Parvoryx01 trial [18].

How might one explain this discrepancy between full permissiveness and susceptibility to H-1PV in vivo and the limited efficacy of H-1PV treatment of the animals? First, rats are the natural host of H-1PV. Thus, a mouse model systematically underestimates the treatment efficacy of H-1PV, since outside the xenograft tumor it does not provide the host cell environment required for virus persistence. Once the tumor shows a reduced proliferation rate or even becomes necrotic, the substrate for virus replication is reduced and consequently the virus is eliminated.

In preclinical testing on glioblastoma [22], and pancreatic ductal adenocarcinoma [23], long-term cure rates were achieved only in rat models. Secondly, provided the virus shows lytic activity in the chosen model, immunocompetent animal models recruiting the immune system of the host for an intensified antitumor immune response are more likely to display sustained, virus-induced tumor control. In each malignant disease for which H-1PV has been tested, immunocompetent animal models have always provided the best response or even cure rates [21]. Thus, future efforts to demonstrate the full therapeutic potential of H-1PV for the treatment of EWS patients should focus on immunocompetent animal models of EWS, which so far are not available to our group. Their future use will avoid underestimating virus-induced immunostimulatory and vaccination effects in immunodeficient animals and will provide data allowing a systematic comparison with other immunotherapeutic approaches currently under preclinical development for Ewing sarcoma patients.

## 5. Conclusions

The data presented here are part of a project seeking promising indications for oncolytic virotherapy based on protoparvovirus H-1 in pediatric cancer patients. Our systematic preclinical analysis confirms the oncoselectivity of H-1PV infection in mesenchymal cells. We show that Ewing sarcoma cells derived from tumors at different stages of development are efficiently infected by H-1PV, sustain viral protein synthesis, and actively replicate fully infectious H-1PV particles. Furthermore, low to moderate doses of input virus are shown to induce cytopathic effects in the four cell lines tested. Although these in vitro data point to Ewing sarcoma as a promising disease for quantifying virus-induced antineoplastic effects in vivo, the antineoplastic effects observed in vivo failed to reach statistical significance. H-1PV as a therapeutic agent used alone thus appears unable to achieve complete and sustained local tumor control in the TC-71 xenograft mouse model used here. Despite its limitations, this animal model should allow testing optimized treatment strategies aiming to increase cytotoxic efficacy against EWS by combining optimized H-1PV treatment with administration of one or several currently used antineoplastic drugs. In immunodeficient xenograft models, the immunostimulatory effects of parvovirotherapy are usually underestimated. Obtaining a clearer picture of the in vivo therapeutic potential of H-1PV in the context of Ewing sarcoma should thus require using a panel of syngeneic immunocompetent animal models. Systematic and comparable preclinical studies on immunocompetent Ewing sarcoma animal models may provide final proof-of-concept in vivo and should allow a precise evaluation of immunological effects in combinatorial treatment approaches. These preclinical evaluation steps are essential prerequisites to preparing well-designed clinical trials evaluating oncolytic parvovirotherapy in children, adolescents, and young adults suffering from Ewing sarcoma.

## Figures and Tables

**Table 1:** Origin of the Ewing sarcoma (EWS) cell lines and ED50 of H-1PV as determined by cytotoxicity testing.

Cell Line	LD50 [PFU/Cell]	Tumor of Origin	Age of Patient
MHH-ES-1	0.01	Peritoneal metastatic EWS	12 years
TC-71	0.1	Local recurrent EWS	22 years
SK-ES-1	5	Primary Ewing sarcoma	18 years
CADO-ES1	5	Pleural metastatic EWS	19 years

LD50 values for Ewing sarcoma cell lines six days after H-1PV infection. The LD50 values were determined on the basis of the observed cytopathic effects, metabolic activity, and lysis of infected cells (MTT test and LDH-release assay) as shown in Figure 4.

**Table 2:** ED50 values for Ewing sarcoma cells with fibroblast-like morphology.

Cell Line	ED50 [PFU/Cell]	*R* ^2^	Observation Time
MHH-ES	0.32	0.922	256 h
SK-ES-1	0.11	0.972	215 h
TC-71	1.04	0.978	120 h

ED50 values for the three Ewing sarcoma cells with fibroblast-like morphology. The ED50 values were determined by real-time proliferation measurements (xCelligence, Roche, Basel, Switzerland). An approximation method using a sigmoid dose-response graph was used as described under Materials and Methods.

## Supplementary Materials

Supplementary materials can be found at http://www.mdpi.com/1999-4915/10/6/302/s1.
