# Supplementary material for: Preclinical Testing of an Oncolytic Parvovirus in Ewing Sarcoma: Protoparvovirus H-1 Induces Apoptosis and Lytic Infection In Vitro but Fails to Improve Survival In Vivo
Source: Viruses. 2018 Jun 3;10(6):302. doi: 10.3390/v10060302 (PMC6024310; doi:10.3390/v10060302)
Supplement: Supplementary file 1 [file viruses-10-00302-s001.pdf]

Supplementary Materials and Figures:

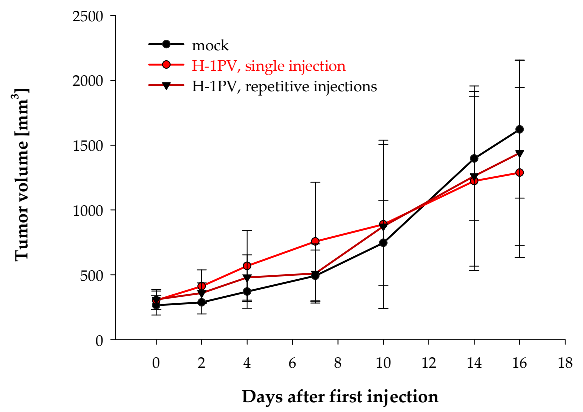

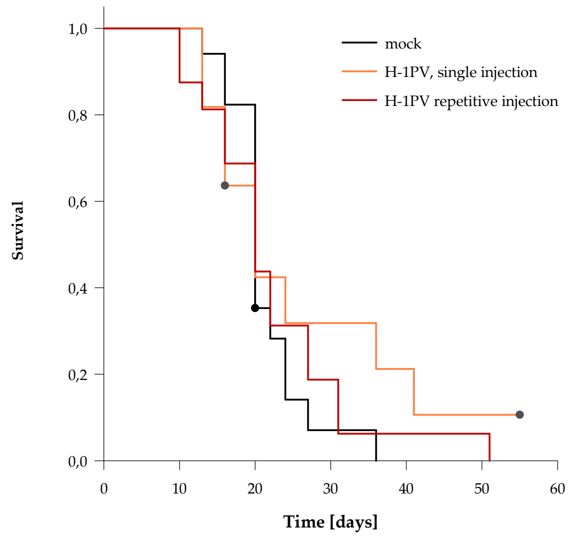

Supplementary Figure 1. H-1PV infection represses growth of subcutaneous TC-71 xenograft tumors in mice. (A) Mean tumor volume of animals after a single intratumoral injection sodium chloride 0.9% control group, n=15, black graph). Animals in the treatment groups (n=15, red graph) received either a single intratumoral injection of 10^9 PFU wild type H-1PV (n=11, orange graph) or repeated intratumoral doses of 10^9 PFU wild type H-1PV twice per week (n=15, dark red graph). For each group of animals the standard errors of the mean are indicated as error bars. (B) Survival data of the three groups of animals (mock-infected, single intra-tumoral H-1PV infection, intra-tumoral H-1PV infection twice a week) do not show statistically significant differences, but indicate two animals in complete long-term remission after H-1PV virotherapy.
